# Thyroid-stimulating immunoglobulin levels during low-dose antithyroid therapy predict Graves’ disease relapse

**DOI:** 10.3389/fendo.2025.1697122

**Published:** 2025-10-29

**Authors:** Phong Vu Nhat Nguyen, Khue Thy Nguyen

**Affiliations:** 1 Department of Endocrinology, School of Medicine, University of Medicine and Pharmacy at Ho Chi Minh City, Ho Chi Minh City, Vietnam; 2 Medic Medical Center, Ho Chi Minh City, Vietnam

**Keywords:** Graves’ disease, relapse, thyroid-stimulating immunoglobulin, treatment duration, thyroid eye disease

## Abstract

**Background:**

Graves’ disease (GD) is a common autoimmune disorder often treated with antithyroid drugs (ATD), but relapse rates remain high after therapy withdrawal. This study evaluates the role of thyroid-stimulating immunoglobulin (TSI) levels in predicting relapse after ATD discontinuation in GD patients.

**Methods:**

A retrospective analysis of 349 GD patients who achieved euthyroid status on maintenance ATD therapy was conducted. TSI levels were measured using a chemiluminescent immunoassay. Of these, 88 patients discontinued ATD therapy and were monitored for relapse. Statistical analyses, including receiver operating characteristic curve analysis, were used to identify relapse predictors.

**Results:**

The median TSI level was significantly higher in patients who relapsed (1.63 vs. 0.52 IU/L, p<0.01), and a cutoff of 1.31 IU/L yielded 63.64% sensitivity, 78.79% specificity, and 86.67% negative predictive value. Independent relapse predictors included a TSI ≥1.31 IU/L, treatment duration of less than 18 months, and the presence of thyroid eye disease.

**Conclusion:**

TSI measurement is a valuable tool for predicting relapse after ATD withdrawal in GD. A TSI cutoff of 1.31 IU/L can aid in clinical decision-making, but further prospective studies are required to confirm these findings.

## 1 Introduction

Graves’ disease (GD), a prevalent autoimmune disorder, is the leading cause of hyperthyroidism, affecting approximately 3% of women and 0.5% of men worldwide over their lifetime (1, 2). This condition is driven by thyroid-stimulating immunoglobulins (TSI), which activate the thyroid-stimulating hormone (TSH) receptor, leading to thyroid hyperplasia and excessive hormone production. Clinically, GD often presents with hyperthyroid symptoms, goiter, and hypervascularization of thyroid parenchyma on Doppler ultrasound, with some patients developing specific autoimmune manifestations such as thyroid eye disease (TED) and dermopathy.

Recent advancements in TSI measurement have introduced the automated bridge chemiluminescent immunoassay, which provides specific detection of stimulating antibodies, distinguishing them from blocking or neutral antibodies. This innovation addresses the limitations of traditional TSH-receptor antibody (TRAb) assays, which measure total TSH-receptor binding inhibitory immunoglobulins (TBII) without differentiating their functional activity (3, 4). The automated TSI assay has shown diagnostic performance that is comparable to, or potentially superior to, TRAb assays, enhancing its utility in diagnosing hyperthyroidism and managing patients with TED (5).

Despite effective treatment with antithyroid drugs (ATDs), relapse rates after medication withdrawal remain high (50%–80%), underscoring the need for reliable predictors of remission and relapse (6, 7). While TRAbs measurement is recommended before ATD withdrawal (8, 9), its limitations in distinguishing antibody subtypes create uncertainty in predicting outcomes. In this context, TSI immunoassays hold promise for more accurate risk stratification, though their clinical utility remains underexplored.

This study was designed to address two specific aims: (1) to evaluate serum TSI concentrations in GD patients receiving maintenance doses of ATDs; and (2) to determine the predictive value of TSI levels for relapse in GD patients after ATD discontinuation. Addressing the aims would provide insights into autoimmune activity during maintenance therapy and the identification of an optimal TSI cutoff value to improve risk stratification and clinical decision-making in the management of GD.

## 2 Materials and methods

### 2.1 Design and patients

This retrospective follow-up study included non-pregnant patients with hyperthyroidism caused by GD, who were treated at a specialized endocrine outpatient clinic in Ho Chi Minh City, Vietnam, between January 2000 and April 2021.

The following inclusion criteria were applied: Patients diagnosed with hyperthyroidism due to GD, as defined by the Japan Thyroid Association criteria (10). Doppler ultrasound was performed during the initial evaluation and prior to considering the discontinuation of treatment to assess thyroid volume and parenchymal vascularity. Clinical assessments of eye signs and symptoms were supplemented with ultrasound or MRI evaluations of the periocular muscles during the same period to support the diagnosis of TED. On MRI, extraocular muscle enlargement was defined as a maximum diameter exceeding 3.5 mm (11). Moreover, patients who underwent TSI measurement during treatment, particularly when medication cessation was being considered, and whose Free Thyroxine (FT4) levels had normalized while receiving maintenance doses of ATDs: Methimazole (MMI) ≤5 mg/day or Propylthiouracil (PTU) ≤100 mg/day.

The following patients were excluded from the study: (1) Patients younger than 18 years at the time of TSI measurement; (2) Patients with hyperthyroidism caused by conditions other than GD; (3) Patients who were intolerant to ATD or experienced significant side effects; and (4) Patients who had undergone surgical treatment or radioactive iodine therapy.

**Figure 1:**
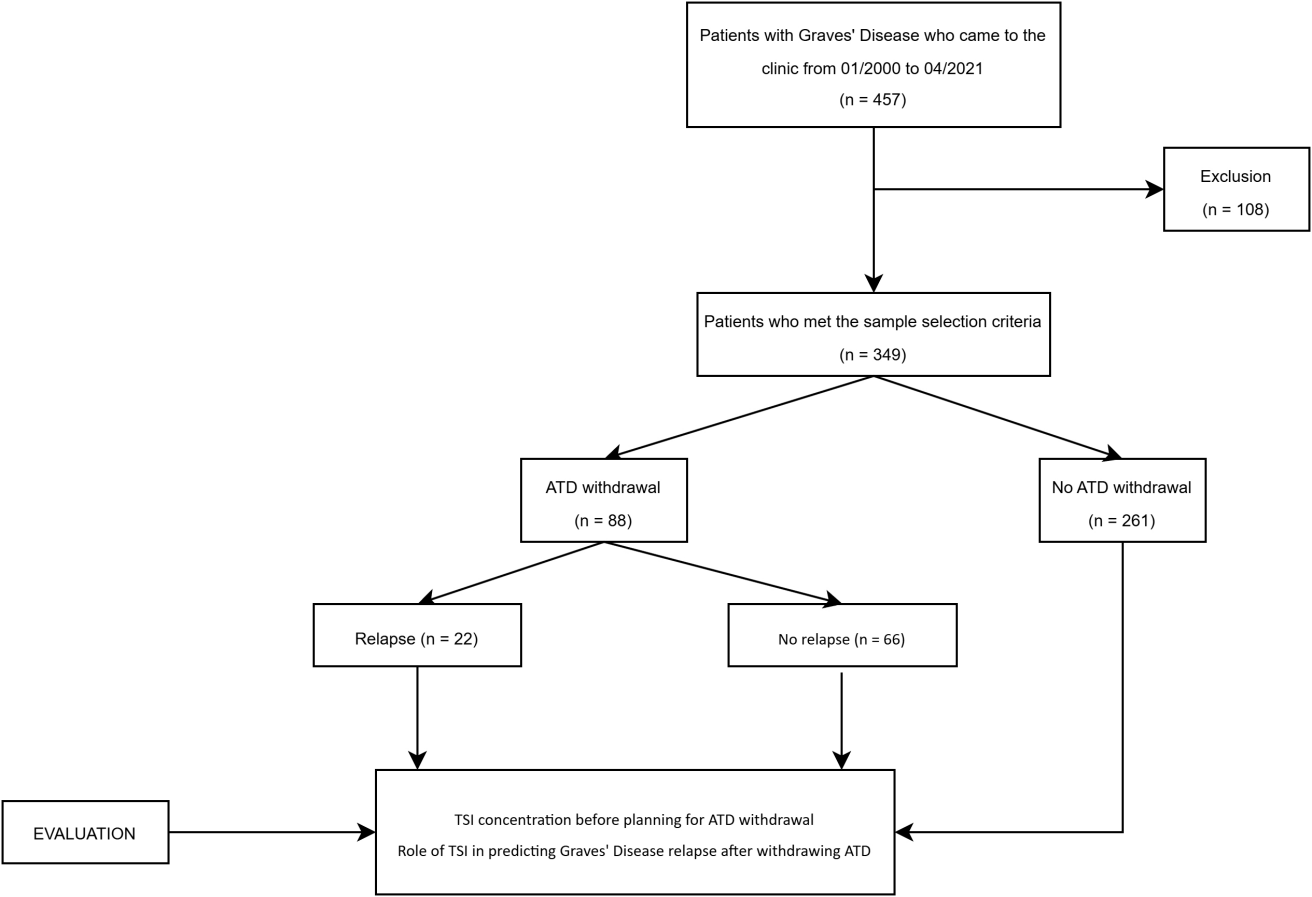
Flowchart of patient selection. ATD, Antithyroid drug.

In total, using the convenience sampling method, we enrolled 457 GD patients and excluded 108 who met the exclusion criteria. During the course of treatment, ATD therapy was discontinued in 88 patients. These patients were further subdivided into two subgroups relapse (n = 22) and no relapse (n = 66) (**Figure 1**). TSI levels were measured prior to considering the withdrawal of ATD. The decision to discontinue ATD therapy was based on an overall clinical evaluation by the attending physician. This evaluation included several factors: the achievement of a clinically euthyroid state, thyroid function tests within normal ranges (with maintenance of normal FT4 levels for at least three consecutive months), stabilization on a minimal dose of ATD (≤5 mg/day of methimazole or ≤100 mg/day of propylthiouracil), TSI concentrations, normalization of vascularity on Doppler ultrasound.

During the maintenance phase of ATD therapy, clinical factors associated with GD relapse were assessed, including age at diagnosis, sex, smoking status, and goiter characteristics. Goiter was defined as diffuse thyroid enlargement, assessed clinically or ultrasonographically. Clinically, it included any palpable and/or visible thyroid enlargement (grades 1 and 2 per WHO criteria) (12). Ultrasonographically, an enlarged thyroid in our center was defined as a total volume exceeding 20 mL. Increased parenchymal vascularity was identified via Doppler ultrasound by detecting diffusely hypervascularity of thyroid parenchyma. TED was evaluated clinically based on characteristic symptoms, such as exophthalmos or periorbital swelling, and confirmed using orbital ultrasound or MRI, where extraocular muscle enlargement was defined in our clinic as a maximum diameter exceeding 3.5 mm.

Smoking status was recorded at the beginning of the study, and no participants reported quitting smoking during the study period.

Following the discontinuation of ATD therapy, patients were monitored at the clinic for a minimum of 12 months to confirm clinical euthyroid and periodically check the thyroid function tests. During this follow-up period, patients exhibiting symptoms and signs indicative of hyperthyroidism or those presenting with suppressed TSH and elevated FT4 levels were considered to have relapse (8).

### 2.2 Laboratory measurements

The automated bridge chemiluminescent assay (IMMULITE 2000, Siemens Healthcare Diagnostics Inc^®^) was utilized for measuring TSI. This assay has a reportable range of 0.10–40.0 IU/L, with a positivity threshold of ≥0.55 IU/L, demonstrating a sensitivity of 100% and specificity of 99%. The assay’s repeatability coefficient of variation (CV) and within-laboratory CVs are ≤7.0% and ≤8.3%, respectively (13).

This method employs a pair of recombinant human TSH receptors (hTSHR) designed in a bridging immunoassay format. The capture receptor, which linked to one arm of the patient’s Thyroid Receptor Antibody (TRAb in the sample), is immobilized on a solid phase (polystyrene bead); the signal receptor is an alkaline phosphatase-labeled recombinant hTSHR in a buffer solution (SEAP-secretory alkaline phosphatase). In the first test cycle, one arm of patient’s TSH receptor autoantibody binds to the capture receptor. In the second cycle, the signal receptor binds to the other arm of patient’s autoantibody, forming a bridge. Finally chemiluminescent substrate is added to the reaction tube. The intensity of enhanced chemiluminescence developed by reaction of secretory alkaline phosphatase with luminescent substrate representing the amount of signal receptor bound, and thus the amount of antibody bound (13).

The IMMULITE 2000 TSI assay was compared to the Thyretain TSI receptor bioassay using 244 serum samples from patients with Graves’ disease and other immune thyroid disorders. The positive agreement rate was 99.22%, with a sensitivity of 98.3% and specificity of 99.7 (14).

Serum levels of TSH and FT4 were measured with the Cobas e602 analyzer (Elecsys, Roche Diagnostics Ltd) with reference ranges for TSH and FT4 of 0.51 – 4.94 mIU/L and 0.71 – 1.85 ng/dL, respectively.

### 2.3 Statistical analysis

Stata software (version 14.0) was used for all statistical analyses. Mean ± standard deviation (SD) or median with interquartile ranges (IQR) were used to present continuous variables, while numbers with proportions were used to present categorical variables. Histogram observations and Kolmogorov-Smirnov test analysis were used to test the normality of distribution for continuous quantitative variables. The Student’s t-test and Mann-Whitney U test were used to compare continuous variables with normal and non-normal distributions between groups (No ATD withdrawal vs. ATD withdrawal groups, Relapse and No relapse groups) respectively. Pearson’s chi-square test was used to compare categorical data.

For the first aim, we assessed the median TSI levels in 349 patients receiving a minimal dose of ATD, along with the corresponding median TSH and FT4 levels.

For the second aim, we evaluated the ability of TSI concentrations measured before ATD withdrawal to predict GD relapse, using the manufacturer-recommended cut-off point of 0.55 IU/L. Additionally, alternative cut-off points were tested to optimize the sensitivity and specificity of TSI levels for relapse prediction through receiver operating characteristic (ROC) curve analysis. To identify predictors of GD relapse, univariate logistic regression analysis was initially performed for each potential variable, including age at diagnosis, smoking status (active or non-smoker at the time of data collection), presence of goiter or TED, increased thyroid parenchymal vascularity, duration of ATD treatment, and TSI levels before ATD withdrawal. Factors with statistically significant associations in the univariate analysis were assessed for multicollinearity before inclusion in the multivariate logistic regression model. Only variables with low multicollinearity were incorporated into the final model. Odds ratios (ORs) and 95% confidence intervals (CIs) were calculated to quantify the strength of associations.

All statistical tests were two-tailed and the level of significance used was 0.05.

## 3 Results

### 3.1 Baseline characteristics of patients

Following data collection, a total of 349 patients were included in the analysis based on our patient selection flowchart (**Figure 1**). Their baseline characteristics are listed in **Table 1**. The mean age at GD diagnosis was 33.22 ± 11.84 years. The majority of the study population (85.96%) was female. TED accounted for 28.08% of the study population. There were no significant differences between two groups (No ATD withdrawal and ATD withdrawal) in term of age, smoking, family history of thyroid disease, duration of GD, enlarged thyroid gland or TED.

**Table 1:** Baseline characteristics of 349 patients stratified by ATD withdrawal.

Characteristics	Total (n = 349)	No ATD withdrawal (n = 261)	ATD withdrawal (n = 88)	p-value
Age at diagnosis (years)	33.22 ± 11.84	33.39 ± 11.89	32.69 ± 11.76	0.62
Female sex, n (%)	300 (85.96)	218 (83.52)	82 (93.18)	0.02
Smoking, n (%)	60 (17.19)	47 (18.01)	13 (14.77)	0.48
Family history of autoimmune thyroid disease, n (%)	78 (22.34)	62 (23.75)	16 (18.18)	0.27
Duration of GD (years)	6 (2 – 13)	6 (3 – 14)	5 (1 – 11.5)	0.06
Enlarged thyroid gland, n (%)	237 (67.90)	183 (70.11)	54 (61.36)	0.13
TED, n (%)	98 (28.08)	69 (26.44)	29 (32.95)	0.23
Increased thyroid parenchymal vascularity, n (%)	197 (56.45)	155 (59.39)	39 (44.32)	0.01
TSI at maintenance dose (IU/L)	1.46 (0.45 – 4.81)	1.89 (0.63 – 5.97)	0.84 (0.26 – 2.37)	<0.01
Positivity of TSI (≥0.55 IU/L), n (%)	254 (72.78)	202 (77.39)	52 (59.09)	<0.01
TSH at maintenance dose (mIU/L)	1.04 (0.04 – 2.12)	0.87 (0.01 – 1.84)	1.66 (0.81 – 2.55)	<0.01
FT4 at maintenance dose (ng/dL)	1.18 (0.97 – 1.43)	1.18 (0.97 – 1.44)	1.16 (0.97 – 1.41)	0.99

Values are expressed as mean ± SD, or median (interquartile range), or number (%).

ATD, antithyroid drug; GD, Graves’ disease; TED, Thyroid eye disease.

TSI, thyroid-stimulating immunoglobulin (Reference range: <0.1 IU/L); TSH, thyroid-stimulating hormone (Reference range: 0.51 – 4.94 mIU/L); FT4, free thyroxine (Reference range: 0.71-1.85 ng/dL).

### 3.2 TSI levels before planning for ATD withdrawal

For the first aim, when assessing the TSI concentration at the maintenance dose of ATD, or in other words, the TSI concentration before planning for ATD withdrawal, we found that the median TSI concentration was 1.46 IU/L, while the thyroid function measured at the same time was euthyroid (**Table 1**). Moreover, TSI levels in the No ATD withdrawal group was significantly higher than in the ATD withdrawal group (**Table 1**).

### 3.3 Characteristics of patients with and without relapse after ATD withdrawal

In the 88 patients who discontinued ATD treatment, the rate of relapse within at least 12 months of follow-up in our study was 25%. According to the clinical outcomes following ATD withdrawal, patients were then divided into two groups, one group without relapse (n=66) and another group with relapse (n=22). Their characteristics are summarized in **Table 2**.

**Table 2:** Data of patients with or without relapse after ATD withdrawal (n = 88).

Patient data	Without relapse (n = 66)	With relapse (n = 22)	p-value
Age at diagnosis (years), mean ± SD	32.90 ± 12.14	32.04 ± 10.77	0.76
Female sex, n (%)	60 (90.91)	22 (100)	0.33
Active smoking, n (%)	11 (16.67)	2 (9.09)	0.50
Family history of autoimmune thyroid disease, n (%)	11 (16.67)	5 (22.73)	0.53
Enlarged thyroid gland, n (%)	38 (57.58)	16 (72.73)	0.20
Thyroid eye disease, n (%)	18 (27.27)	11 (50)	0.05
Increased thyroid parenchymal vascularity, n (%)	26 (39.39)	13 (59.09)	0.10
Euthyroid duration under ATD (months)	22.5 (16–35)	11.5 (8–23)	<0.01
Treatment duration of ATD (years)	2.71 (1.83–4.08)	2.29 (1–2.75)	0.02
Treatment duration of ATD < 18 months	6 (9.09)	9 (40.90)	<0.01
❖ Thyroid-related biochemical tests before ATD withdrawal:			
TSH (mIU/L)	1.86 (1.12–2.40)	2.18 (0.98–2.93)	0.63
FT4 (ng/dL)	1.17 (0.96–1.34)	1.10 (0.95–1.34)	0.71
TSI (IU/L)	0.52 (0.15–1.21)	1.63 (0.37–3.75)	<0.01
Positivity of TSI (≥0.55 IU/L)	31 (46.97)	15 (68.18)	0.08

Values are expressed as mean ± SD, or median (interquartile range), or number (%).

ATD, antithyroid drug; TSI, thyroid-stimulating immunoglobulin (Reference range: <0.1 IU/L); TSH, thyroid-stimulating hormone (Reference range: 0.51 – 4.94 mIU/L); FT4, free thyroxine (Reference range: 0.71-1.85 ng/dL).

For the second aim, which focused on TSI concentration before ATD withdrawal as a potential predictor of relapse, the relapse group had a significantly higher TSI concentration compared to the no-relapse group (1.63 vs. 0.52 IU/L, p<.01). However, when applying the manufacturer’s cut-off value of 0.55 IU/L, there was no significant difference in the TSI positivity rate between the two groups (**Table 2**). Other covariates, such as age at diagnosis, gender, smoking status, family history of thyroid disease, the presence of an enlarged thyroid gland, increased thyroid parenchymal vascularity, TED rate, and thyroid function parameters (including TSH and FT4) at the end of treatment, did not show significant differences between the groups. Notably, the no-relapse group had a significantly longer euthyroid duration under ATD, and a longer overall treatment duration (22.5 vs. 11.5 months, p<.01 and 2.71 vs. 2.29 years, p=.02, respectively). Additionally, the relapse group had a significantly higher rate of ATD treatment duration below 18 months (**Table 2**).

### 3.4 TSI level cut-off to predict Graves’ disease relapse

**Figure 2:**
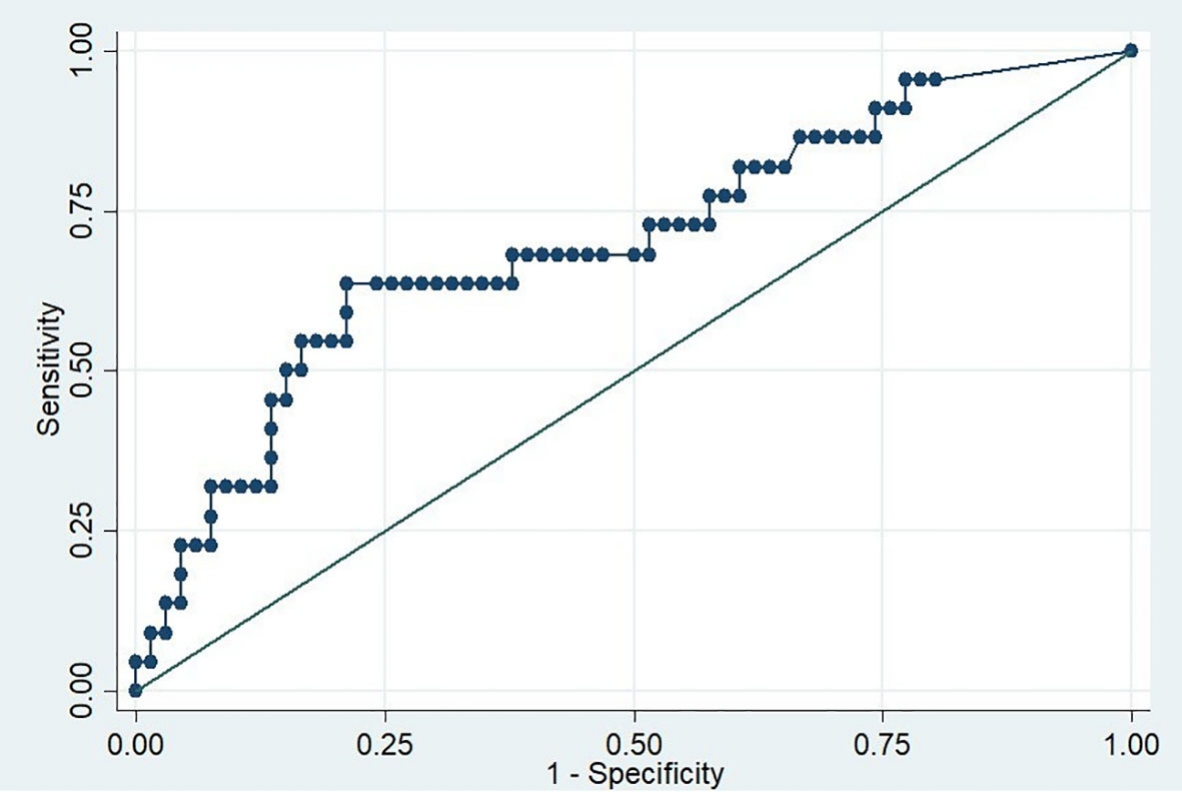
ROC curve for predicting relapse of Graves’ disease based on TSI levels before antithyroid drug withdrawal.

For the second aim, since the TSI cut-off value recommended by the manufacturer (0.55 IU/L) did not show a statistically significant difference between the two groups, we sought to identify an optimal cut off to predict relapse of GD after medication withdrawal. Using the ROC curve, we calculated the Area Under the Curve (AUC) of 0.71 (95% CI, 0.60 – 0.83) (**Figure 2**) and identified the optimal cut-off point of 1.31 IU/L using the maximum Youden’s index, which balances sensitivity and specificity. At this cut-off point, the sensitivity, specificity and the percentage correctly classified were 63.64%, 78.79%, and 75%, respectively. In addition, the calculated negative predictive value of this cut-off was 86.67%, indicating that if TSI level before ATD withdrawal is less than 1.31 IU/L, 86.67% of the patients will remain in remission after discontinuing the medication for at least 12 months (**Table 3**).

**Table 3:** Diagnostic values for TSI level cut-off point of 1.31 IU/L prior to withdrawal of ATD.

Diagnostic metric	Point estimate (95% CI)
Sensitivity	63.64 (40.70 – 82.80)
Specificity	78.79 (67.00 – 87.90)
Positive predictive value (PPV)	50.00 (30.60 – 69.40)
Negative predictive value (NPV)	86.67 (75.40 – 94.10)
Percentage correctly classified	75.00 (64.60 – 83.60)

ATD, antithyroid drug; CI, confidence interval.

### 3.5 Factors associated with GD relapse

Building on the second aim, univariate and multivariate logistic regression analyses were conducted to further explore factors associated with relapse in the ATD withdrawal group. These analyses also examined the potential of a TSI cut-off level of 1.31 IU/L prior to ATD withdrawal as an independent predictor of GD relapse risk. In univariate logistic regression analysis, we found that a shorter time spent in euthyroid prior to ATD discontinuation, treatment duration of less than 18 months, and TSI levels of ≥1.31 IU/L prior to ATD withdrawal were all significant predictors of relapse in GD patients. Subsequently, the multivariate logistic regression analysis revealed that three following factors were independent predictors of GD relapse risk: the presence of TED (with an OR of 3.69), a treatment duration of less than 18 months (with an OR of 7.77), and TSI levels of ≥1.31 IU/L before ATD withdrawal (with an OR of 9.92) (**Table 4**).

**Table 4:** Univariate and multivariable logistic regression analysis for predicting Graves’ disease relapse.

Patient data	Univariate analysis		Multivariable analysis	
	OR (95% CI)	p-value	OR (95% CI)	p-value
Age at diagnosis (years)	0.99 (0.95-1.03)	0.76		
Smoking (yes)	0.50 (0.10-2.45)	0.39		
Enlarged thyroid gland (yes)	1.96 (0.68-5.65)	0.21		
Thyroid eye disease (yes)	2.66 (0.98-7.21)	0.05	3.69 (1.06-12.87)	0.04
Increased thyroid parenchymal vascularity (yes)	2.22 (0.83-5.93)	0.11		
Euthyroid duration under ATD (months)	0.94 (0.90-0.98)	0.01		
Treatment duration of ATD < 18 months (yes)	6.92 (2.09 – 22.85)	<0.01	7.77 (1.92-31.42)	<0.01
❖ TSI tests before ATD withdrawal:				
TSI (IU/L)	1.08 (0.99 – 1.17)	0.08		
Positivity of TSI (≥0.55 IU/L) (yes)	2.41 (0.87-6.70)	0.08		
Positivity of TSI (≥1.31 IU/L) (yes)	6.50 (2.27-18.57)	<0.01	9.92 (2.77-35.42)	<0.01

CI, confidence interval; OR, odds ratio; ATD, antithyroid drug;

TSI, thyroid-stimulating immunoglobulin (Reference range: <0.1 IU/L); TSH, thyroid-stimulating hormone (Reference range: 0.51 – 4.94 mIU/L); FT4, free thyroxine (Reference range: 0.71-1.85 ng/dL).

## 4 Discussion

TSI remains so far, the most specific biomarker of GD. However, the detection of TSI was previously limited to bioassays, which were complex, expensive and time consuming, making it challenging to incorporate them into routine laboratory procedures, especially in low-middle income countries. While TSI and other indicators have been studied previously, our study aimed to contribute additional insights by applying an automated TSI immunoassay in a population-specific context, particularly in Vietnam, where data on GD management remain limited. Furthermore, Vietnam remains classified as iodine insufficient according to the Iodine Global Network 2025 Scorecard (15). This iodine status may affect relapse rates in GD, as evidence from systematic reviews indicates that adequate iodine intake is associated with lower recurrence rates, higher remission rates, and greater efficacy in managing thyrotoxicosis compared to insufficient, above-requirement, or excessive iodine levels (16).

In recent years, an automated TSI assay that can directly measure TSI levels has been developed and has shown great promise in preliminary studies for its clinical value (5, 17). Our results showed that, even after reaching euthyroid with low-dose ATD, the autoimmune activity associated with GD may persist, as indicated by the high levels of TSI in the follow-up group of patients. Our findings are consistent with those of De Bellis A, where TRAb remained positive in patients who achieved euthyroid with ATD treatment (18). Additionally, a previous prospective study found that TSI was a valuable biomarker that could indicate the activity level, severity, and/or systemic involvement of GD patients (19).

Patients with higher relapse rate may be associated with several factors, including a high concentration of TSI prior to the ATD withdrawal (8, 9). In our study, patients who relapsed had higher TSI titers prior to ATD cessation compared to those who did not experience relapse, reinforcing the role of TSI as a predictor of relapse risk. Unlike TBII assays, which measure antibodies that inhibit TSH binding, TSI immunoassays specifically detect stimulating antibodies, offering greater specificity for assessing disease activity (5, 20). This specificity aligns with findings from prior studies that demonstrated the superiority of TSI bioassays over TRAb immunoassays in predicting GD relapse (21–23). For instance, in a five-year prospective study in 2012 conducted by Giuliani et al., the use of TSI bioassay (Thyretain Mc4 assay) demonstrated better predictive value and sensitivity for relapse compared to TRAb immunoassays (21). In 2016, a retrospective study by Kwon et al. demonstrated that the TSI bioassay at withdrawal from ATD could predict relapse of GD, but the TRAb assays could not (22). Recently, a retrospective study by Da Silva Santos T et al. also showed that TSI immunoassay is a better predictor of GD relapse after ATD withdrawal compared with TRAb immunoassays (23). These studies all used the manufacturer’s cut-off of TSI assays to predict GD relapse.

We observed that some patients with positive TSI (using the manufacturer’s cut-off of 0.55 IU/L) did not experience relapse during the follow-up period. Moreover, the use of this cut-off did not result in a statistically significant difference between the relapse and no relapse groups. This motivated us to analyze whether another cut-off point could improve the performance of the test. Among the different cut-off points that were tested, the one that exhibited the optimal diagnostic value was 1.31 IU/L. By using this cut-off, 86.67% of patients with TSI levels below 1.31 IU/L were expected to achieve remission after discontinuation of medication. Our proposed cut-off is in is closely aligned with the one suggested by Fontes R et al. (1.11 IU/L) for predicting GD relapse (24).

Concerning other risk factors for relapse, in our study, we identified three independent factors that were associated with GD relapse after medication withdrawal: the presence of TED, treatment duration of less than 18 months, and TSI levels equal to or greater than 1.31 IU/L before ATD withdrawal.

The association between TED and a higher risk of hyperthyroidism relapse has been suggested due to the close association of the thyroid gland and the eye as targets of TSI. In our study, the presence of TED was significantly associated with relapse after ATD withdrawal, with an OR of 3.69 (95% CI: 1.06–12.87). This result aligns with findings from previous studies, including those by Vitti et al. and Eckstein et al. (7, 25). These findings highlight the need for closer monitoring of patients with TED following ATD withdrawal, as they are at a higher risk of relapse.

Regarding the treatment duration, a Korean study found that the longer ATD was used, the lower the relapse rate in GD (26), while most thyroid associations recommend treating GD with ATD for at least 12–18 months (8, 9). In our study, treatment duration of less than 18 months was associated with an increased risk of relapse. This result is consistent with previous findings that longer-term ATD treatment increases remission rates (27–29).

Regarding the TSI level prior to medication cessation, our study found that this factor is a significant predictor of GD relapse, with an optimal cut-off value of 1.31 IU/L. This finding is also consistent with recent clinical trials that have shown TSI to be a more specific marker for predicting GD relapse compared to TRAb assays (23, 24).

These findings should be considered within potential weaknesses. As a retrospective analysis, the data could have inherent bias by residual confounders. The sample size was relatively small, which might affect the robustness of the findings. Moreover, the follow-up duration for some patients may have been insufficient to capture late relapses, particularly during the COVID-19 pandemic, when follow-up adherence was disrupted. Nonetheless, this study is among the first to evaluate the predictive value of TSI immunoassays in a Vietnamese population and offers critical insights into their clinical application. By establishing a practical TSI cutoff for relapse prediction, our findings contribute to advancing the management of GD, providing a reliable tool for clinicians to balance treatment efficacy and patient safety.

## 5 Conclusion

These data suggest that TSI levels measured using immunoassays may be a valuable tool for predicting GD relapse after ATD withdrawal. However, these results should be interpreted with caution due to the study’s retrospective design, small sample size, and the discretionary nature of ATD discontinuation. A prospective study with a larger cohort is necessary to validate these findings and further refine the clinical utility of TSI as a predictive biomarker.

## Data Availability

The data analyzed in this study is subject to the following licenses/restrictions: The data analyzed in this study is subject to the following licenses/restrictions: Due to its ethical concerns, supporting data cannot be made openly available. Further inquiries can be directed to the corresponding author. Requests to access these datasets should be directed to Phong Vu Nhat Nguyen. Requests to access these datasets should be directed to nhatphong@ump.edu.vn.
